# Impact of Anticoagulant and Antiplatelet Therapy on the National Optimal Lung Cancer Pathway (NOLCP)

**DOI:** 10.7759/cureus.92169

**Published:** 2025-09-12

**Authors:** Alexandra Jagla, Cyrus Daneshvar, Helen McDill, Maged Hassan

**Affiliations:** 1 Department of Respiratory Medicine, University Hospitals Plymouth NHS Trust, Plymouth, GBR; 2 Department of Respiratory Medicine, Derriford Hospital, Plymouth, GBR; 3 Department of Respiratory Medicine, Southmead Hospital, Bristol, GBR; 4 Department of Chest Diseases, Alexandria University Faculty of Medicine, Alexandria, EGY

**Keywords:** anticoagulation, clinical research work, lung cancer, lung cancer diagnosis, nolcp

## Abstract

Background: Lung cancer remains the leading cause of cancer-related mortality in the UK, with early diagnosis critical to improving survival. The National Optimal Lung Cancer Pathway (NOLCP) aims to streamline diagnosis and treatment, recently introducing a three-day direct-to-biopsy target for select patients. However, many lung cancer patients are on medications that increase the risk of bleeding (RoB), which may delay biopsy scheduling.

Methods: A retrospective study was conducted at University Hospitals Plymouth NHS Trust over nine months, evaluating patients who underwent diagnostic procedures leading to a lung cancer diagnosis. Data were collected on referral times, diagnostic intervals, and RoB medication use. Statistical analysis assessed the impact of RoB medications and other factors on time to biopsy.

Results: Of 177 patients undergoing diagnostic procedures, 32 (18%) were on RoB medications, most commonly anticoagulants for atrial fibrillation. The median time from referral to biopsy was 13 days, with only five (15%) meeting the five-day target. While RoB medications did not significantly affect time from referral to biopsy, they did impact clinic-to-biopsy intervals (median 8 vs. 5 days; p=0.022). Only nine (28%) of RoB patients underwent biopsy within five days of clinic, versus 65 (50%) of others who had a final diagnosis of lung cancer. Delays were amplified in patients seen later in the week.

Conclusion: RoB medications were associated with meaningful delays from clinic to biopsy, challenging NOLCP’s three-day biopsy target. Early identification and proactive management of these patients at triage, along with flexible procedural scheduling, are essential to reduce delays and improve diagnostic timelines.

## Introduction

Lung cancer is the third most common cancer in the UK and remains the leading cause of cancer mortality in the UK, accounting for 21% of all cancer deaths [1]. In 2023, 37,750 people in the UK were diagnosed with lung cancer, of which 37% were diagnosed with stages 1 and 2 cancer. As a result of increasing diagnoses of early-stage lung cancer, one-year survival rates have increased to 50% from 46% in 2022 [2]. Despite this, lung cancer still has a relatively low survival rate compared with other cancers. This has been attributed to delays in timely diagnosis and access to treatments and care [3]. Supporting this, data from a multi-centre randomised controlled trial from six UK centres has shown that reducing the diagnostic pathway from 30 days to 14 days significantly improved the overall median survival [4]. The National Optimal Lung Cancer Pathway (NOLCP) was introduced in 2017 and updated in 2024 to reduce delays from referral to diagnosis to 28 days and treatment from 62 days to 49 days [5]. Following this, programmes such as Rapid Access to Pulmonary Investigations and Diagnosis (RAPID) in Manchester have demonstrated success, with 90% of patients having their CT and consultation within seven days of referral [6]. However, it is recognised that many trusts still struggle with this target due to resourcing, as identified in the National Clinical Leadership Association (NCLA) 2025 report. This showed that only 23% of patients with non-small cell lung cancer (NSCLC) started systemic anti-cancer therapy, and 11% had surgery within the NOLCP target timeframe [2].

The NOLCP previously recommended that biopsies should be performed by day 5 of the pathway. However, the recently updated 2024 guidelines advise a direct-to-biopsy approach within three days for patients who do not require additional staging investigations after triage [5]. Many of these patients have complex comorbidities such as ischaemic heart disease (IHD) and atrial fibrillation (AF), compared with those at risk of other types of cancer, and will be taking medication that increases the risk of bleeding (RoB) [7]. These RoB medications, such as antiplatelet drugs and anticoagulation, may impact the time to biopsy and pathway performance indicators.

Given the complexity of managing these medications during invasive procedures such as biopsies, this study aimed to define the prevalence of RoB-associated medications in our patients and explore the impact this has on our lung cancer service and the feasibility of a three-day direct-to-biopsy approach in the NOLCP.

## Materials and methods

Study site and clinical service

At the University Hospitals Plymouth NHS Trust, where 400 lung cancers are diagnosed annually, a model of care based on the NOLCP has been evolving since 2018. Referrals from the community may come through direct access to chest radiography with same-day thoracic CT scans. Standard suspected two-week wait (2WW) cancer referrals may come through in parallel, along with upgrades from internal sources. Triaging occurs post-CT imaging; for those coming through the direct access route, this occurs the following day. Referrals are seen, if indicated, in daily dedicated lung cancer clinics.

Invasive diagnostic investigations are processed through our patient investigation unit (bronchoscopic procedures) or through our ultrasound service (thoracocentesis, cervical node biopsies, pleural and lung biopsies) - performed by our respiratory physicians, with patients having access to the same-day clinic and diagnostic procedures. Other procedures are performed by interventional radiology, such as image-guided procedures (CT-guided biopsies). The clinical workload is supported by five consultants (working 1.5 whole-time equivalent (WTE)) and three interventional fellows.

Data collection

This retrospective cohort included all patients referred to our service who were subsequently diagnosed with a new cancer over a nine-month period. No specific inclusion or exclusion criteria were applied. Patients were identified using existing tracking tools, which were used to determine timelines between referral, CT imaging, clinics, and diagnostic procedures.

RoB medications

Clinic letters were reviewed to establish whether RoB medications were prescribed and to record the clinical indications. RoB medications were defined as (i) antiplatelet agents clopidogrel, ticagrelor and prasugrel, which typically need to be held for five days; and (ii) direct oral anticoagulants (DOACs) apixaban and rivaroxaban, requiring to be held for 48 hours and warfarin requiring international normalised ratio (INR) checking. Aspirin was not considered to have an impact on sampling times unless taken alongside dipyridamole.

Statistical analysis

The dataset was analysed using Excel (Microsoft® Corp., Redmond, WA, USA) and IBM SPSS Statistics for Windows, Version 26 (Released 2020; IBM Corp., Armonk, New York, United States). Continuous data are presented as median and interquartile range (IQR), and categorical data as frequencies and proportions. Medians were compared using the Wilcoxon rank sum test. Proportions were compared using Fisher’s exact test. Univariate and multivariate regression were used to explore differences in time to procedure, focusing on factors including RoB medications and the day of the week of the patients' first clinic visit. A p-value of <0.05 was considered significant.

## Results

**Figure 1:**
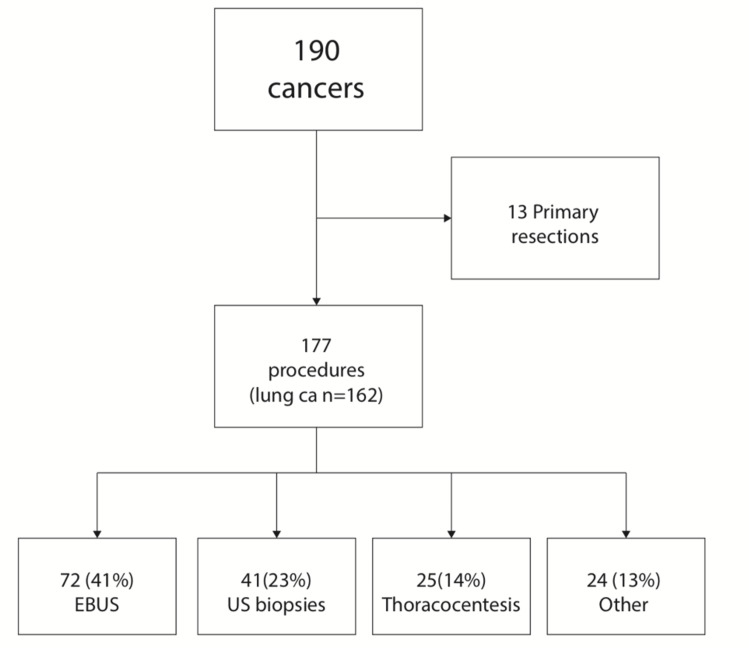
Flow chart demonstrating the number of procedures performed and the ultimate diagnosis of lung cancer. EBUS: endobronchial ultrasound; US biopsies: ultrasound-guided biopsies including neck, pleural and lung; Other: CT-guided biopsies or endobronchial washings, brushing or biopsies

Over a period of nine months, we performed 190 procedures that led to an ultimate diagnosis of malignancy. The median age of this cohort was 71 (range 27-91) years, and 103 of 190 (54%) were male. Of these patients, 162 of 190 (85%) had a final diagnosis of lung cancer. In 13 cases of the 190, non-invasive investigations led directly to primary surgical resection. In the remaining 177, we performed diagnostic procedures. The most common first procedures were endobronchial ultrasound (EBUS), ultrasound-guided biopsies performed by respiratory physicians, and thoracocentesis, in 72 patients (41%), 41 patients (23%), and 25 patients (14%), respectively (Figure 1).

**Figure 2:**
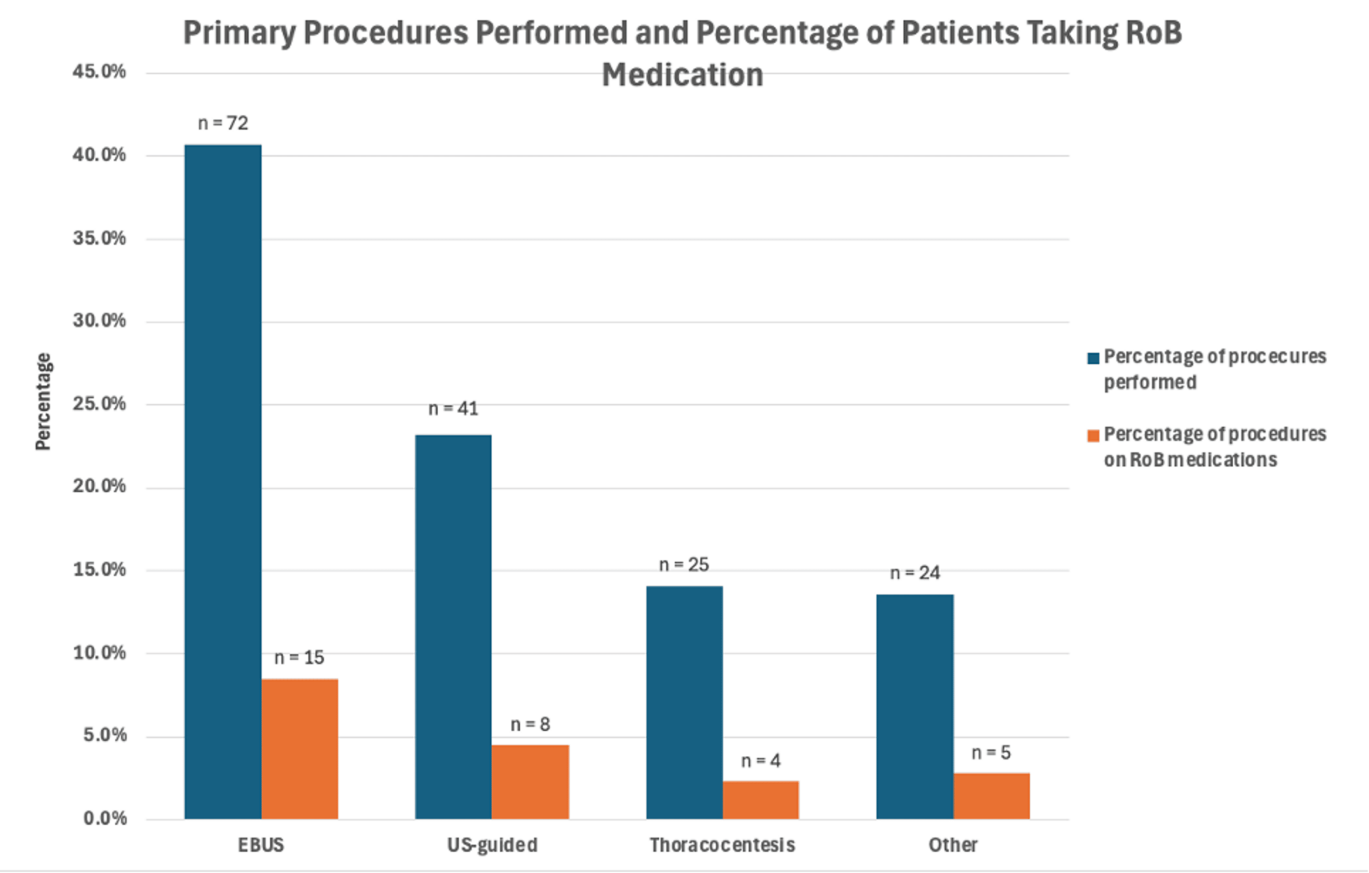
Comparison of the total number of procedures performed with the number of procedures performed on those taking RoB medication. EBUS: endobronchial ultrasound; US biopsies: ultrasound-guided biopsies including neck, pleural and lung; Other: CT-guided biopsies or endobronchial washings, brushing or biopsies

Of those undergoing procedures, 32 of 177 (18%) patients were on RoB medications. The commonest RoB medications prescribed were antiplatelet inhibitors (clopidogrel, prasugrel, dipyridamole or ticagrelor) in 13 (41%) patients, while 19 (59%) of patients were on anticoagulants (DOAC n=11; warfarin n=8). AF and IHD were the commonest indications in 16 (50%) and six (19%), respectively, for use of these medications (Figure 2).

**Figure 3:**
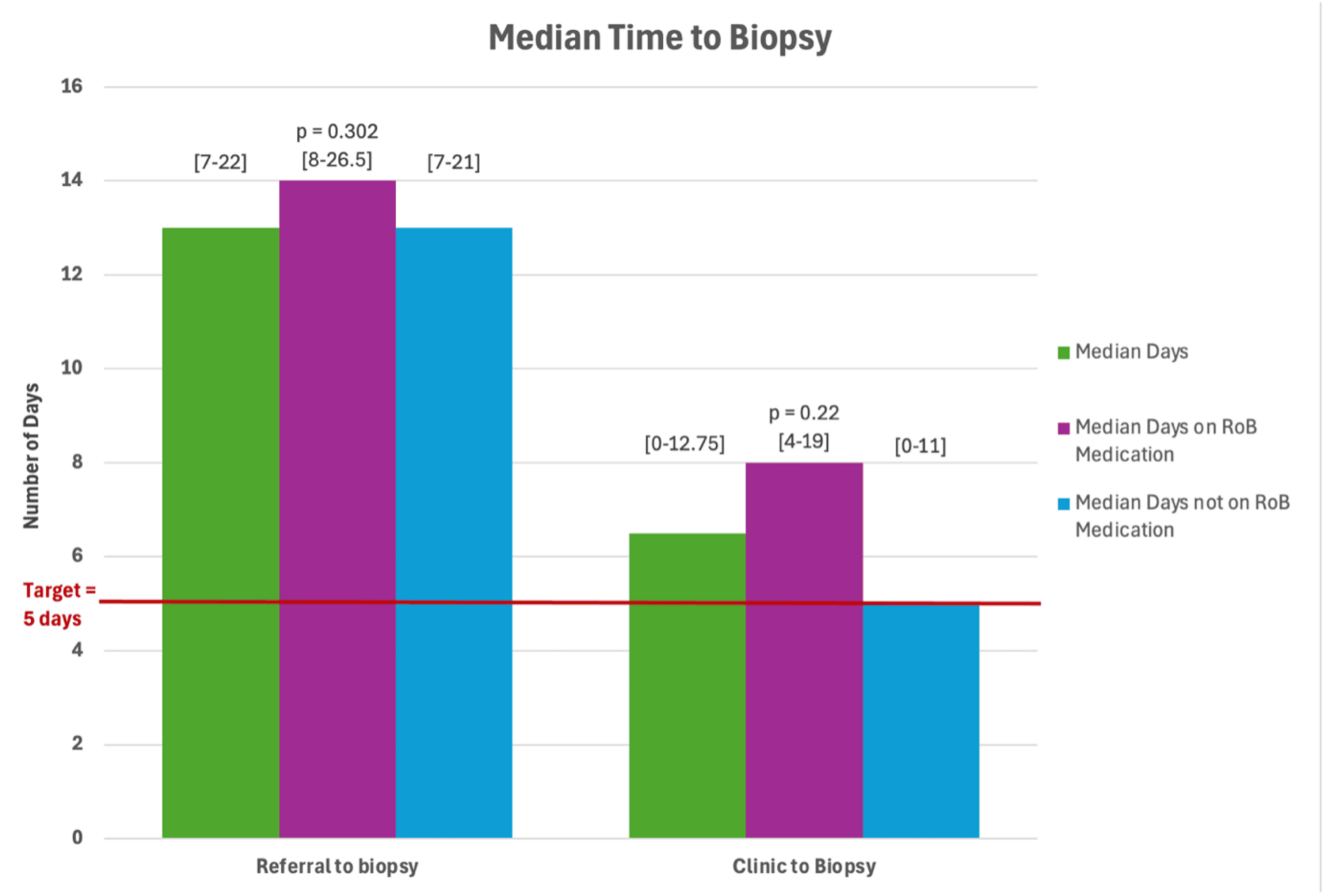
Graph comparing time from referral to biopsy versus clinic to biopsy (median days and IQR) in patients taking RoB medications and those not taking such medications. IQR: interquartile range; RoB: risk of bleeding

The median (IQR range) time from referral to undergoing a biopsy was 13 (7-22) days, with only 24 (15%) achieving the target of sampling within five days. Patients taking RoB medications were not significantly delayed between referral to biopsy compared with those not taking RoB medications (time to biopsy from referral of five days or fewer: 3 (9%) versus 22 (17%) (p=0.224); median 14 (8-26.5) versus 13 (7-21) days (p=0.302)) (Figure 3).

However, 75 (46%) of procedures were performed within five days of clinic with a median time from clinic to biopsy of 6.5 (0-12.75) days. The time from clinic to biopsy was significantly greater in patients on RoB medications compared with those not taking RoB medications (clinic to biopsy: median 8 (4-19) versus 5 (0-11) days; p=0.022). The proportion of patients achieving a time to procedure from clinic of five days or fewer was significantly less in those taking RoB medications (time to biopsy from clinic five days or fewer: 9 (28%) versus 65 (50%) (p=0.018)) (Figure 3).

**Figure 4:**
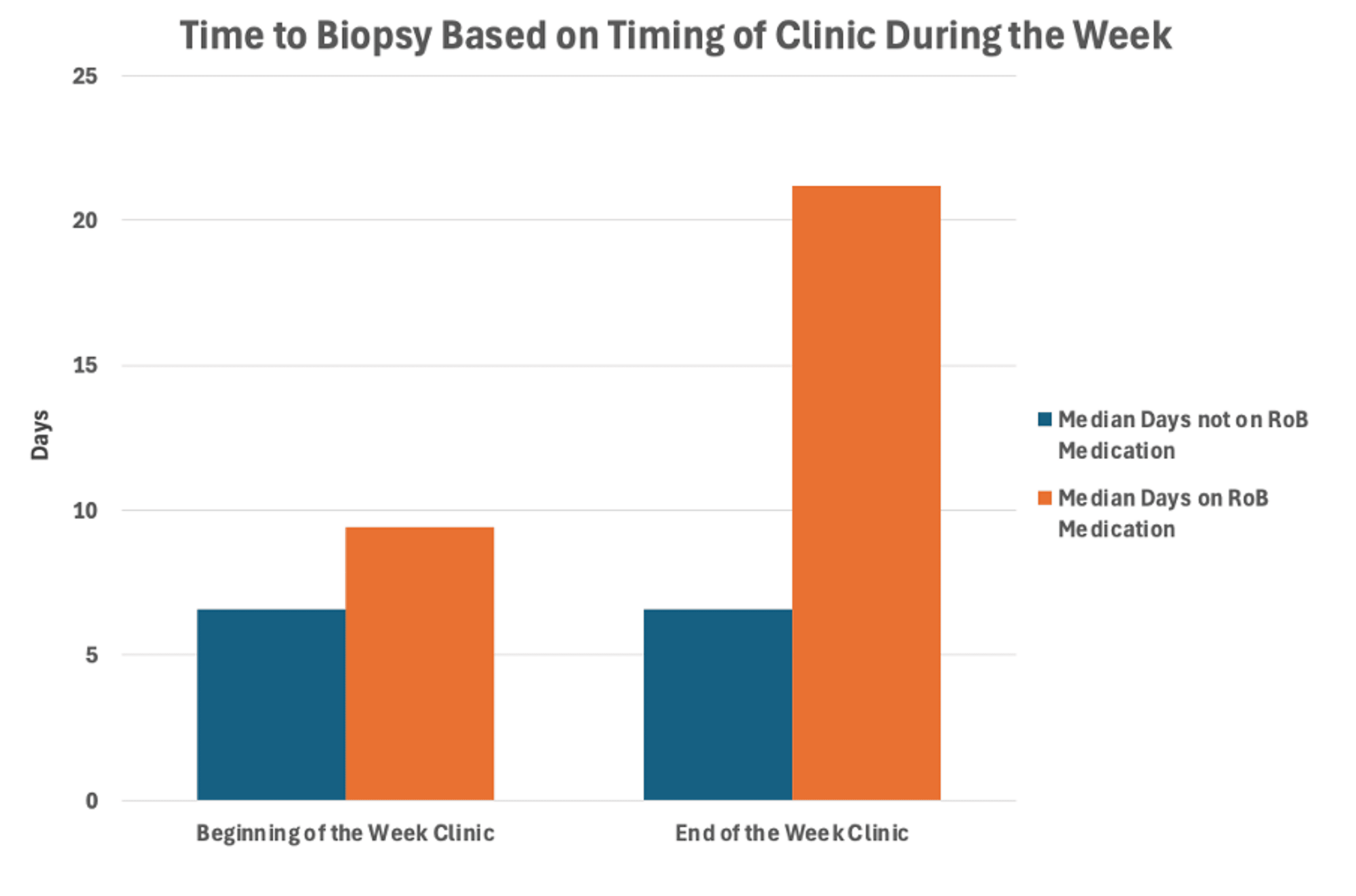
Graph comparing differences in time to biopsy based on timing of referral and whether patients were taking RoB medications. RoB: risk of bleeding

In order to explore factors that may confound these findings, we performed multivariate regression modelling, looking at procedures involving sedation and factors including the timing of referral, CT or clinic appointment. Factors that were associated with a delay from the index CT scan to biopsy included RoB medications (p=0.009), but not the timing of the referral, CT or clinic being at the beginning or end of the week (p=0.21, 0.33 and p=0.99, respectively). When looking at all patients (those on and off RoB medications), we found that there was no significant difference between those seen in a clinic at the beginning of the week (Monday or Tuesday) and the end of the week (Wednesday, Thursday or Friday), with a median wait of 6.6 days. However, those taking RoB medications experienced a median wait of 9.4 days when seen at the beginning of the week, and a further delay to 21.2 days when seen at the end of the week (Figure 4). A univariate analysis also showed that referral to biopsy was not impacted by the timing of referral, CT or clinic (p=0.11, 0.48 and 0.15, respectively).

## Discussion

Medications associated with RoB medications include antiplatelet and anticoagulant therapies, which are primarily used to prevent and treat cardiovascular disease and acute strokes. With around 1.4 million people in the UK suffering from AF, 2.3 million people with coronary heart disease and 100,000 people diagnosed with acute strokes each year, anti-thrombotic drugs are some of the most commonly prescribed medications worldwide [8,9]. Managing these medications during invasive diagnostic procedures can be complex, as guidance varies depending on the drug or combination of drugs used, and established guidance is often outpaced as novel agents are developed.

The British Thoracic Society (BTS) recommends stopping both clopidogrel and warfarin for five days prior to flexible bronchoscopy [10]. The BTS also advises that non-urgent pleural aspirations and chest drains should be avoided in anticoagulated patients until INR <1.5, although it does not specify time periods for holding anticoagulation [10]. Similarly, the American Journal of Roentgenology guidelines advise holding anticoagulation and antiplatelet medication in adults undergoing all percutaneous interventions. For medium bleeding risk interventions, warfarin should be held for five days, DOACs for 48 hours, and antiplatelet agents for five days [11]. This can impact the time to perform diagnostic procedures.

Time to treatment in patients with suspected lung cancer improves patient outcomes, and obtaining a histological diagnosis is one of the first steps in this process [12]. Organisational factors significantly impact the delivery of lung cancer services, with increased resources correlating with a faster pathway to treatment. A growing body of evidence suggests that faster diagnostic pathways lead to higher rates of curative-intent treatment and improve lung cancer survival [13]. This emphasises the importance of pathways redesigns such as the RAPID programme and the NOLCP, which aim to facilitate next-working-day CT scans, immediate "hot" reporting of CT images, and same-day consultations with diagnostic specialists for patients with suspected lung cancer [5,6]. This is supported by the LungBOOST trial, which demonstrated that patients who underwent EBUS staging, compared to conventional staging at the time, experienced a shorter time to diagnosis (14 days versus 29 days) and an improved median survival (312 days versus 503 days) [4].

In our cohort, we found that one in five patients was taking medications that impacted the time to biopsy, with delays ranging from 48 hours to five days. Over half of these patients were taking anticoagulants. AF was the commonest indication for anticoagulation, affecting 16 (50%) of our cohort, followed by IHD in six (19%). While the prevalence of AF in the general population is 2.5%, it was significantly higher in our cohort at 16 (9.6%), suggesting a greater impact on time to biopsy in our cohort than would be experienced in the general population undergoing other procedures [13]. Similarly, the prevalence of IHD is 3.4% in the greater population compared to 10 (6.4%) in our biopsy cohort; however, not all patients with IHD will be taking antiplatelet medications. Therefore, the high prevalence of anticoagulation use in our patients should be an important consideration during triaging for diagnostic procedures within the lung cancer pathway.

In our cohort, the median time from referral to biopsy was 13 days, with only 24 (15%) meeting our target of five days or fewer. Although there was no statistically significant difference in delays from referral to biopsy between patients on and off RoB medications, the time from clinic to biopsy revealed a significant disparity. Overall, 75 (46%) of diagnostic procedures were performed within five days of the clinic, with a median time of 6.5 days. However, for patients taking RoB medications, the median time increased to eight days, with only nine (28%) meeting the five-day target, indicating that RoB medications have a more significant impact following referral rather than the initial scheduling phase. This again highlights the need to routinely identify RoB medications at triage.

At Plymouth NHS Trust, a dedicated pathway co-ordinator is employed and aims to identify RoB medications at triage by reviewing general practitioner (GP) records, then co-ordinating with patients and GPs to either stop or bridge RoB medications prior to clinic, to facilitate immediate diagnostic sampling if indicated. However, limitations remain in stopping RoB medications, as not all RoB medications can be safely discontinued without careful consideration of the underlying medical condition, which may require multidisciplinary input and additional planning or adding bridging therapies. A further limitation is the day of the week on which patients have their CT or clinic appointment, as we found that those taking RoB medications who were referred at the end of the week had longer delays in time to biopsy, with waiting times more than double compared with those seen at the beginning of the week. Additionally, our three-day target pathway must take into account the limitations of the single-centre, retrospective data and small RoB subgroup.

The type of procedure is important and, in our cohort, EBUS was the most common first procedure, followed by respiratory physician-led US biopsy and thoracocentesis. The infrequency and inflexibility of endoscopic procedure lists involving sedation may lead to further delays in patients taking RoB medications. In contrast, physician-led biopsies and thoracentesis are performed more flexibly and can help to reduce delays.

## Conclusions

In one in five patients in our cohort, RoB medications added nearly a one-week delay to biopsy. Early recognition, coordination and linkage of these patients at triage with early decision making on appropriate sampling will mitigate this risk. Targeting a biopsy in 75% of patients within five days would seem a reasonable target, with a focus on immediate early sampling for patients not needing additional tests to achieve the new three-day target.
